# PTPN18 Serves as a Potential Oncogene for Glioblastoma by Enhancing Immune Suppression

**DOI:** 10.1155/2023/2994316

**Published:** 2023-02-15

**Authors:** Tao Wang, Yang Yu, Xinlei Ba, Xiaonan Zhang, Na Zhang, Guowen Wang, Bin Bai, Tong Li, Jiahui Zhao, Yanjiao Zhao, Bing Wang

**Affiliations:** ^1^College of Life and Health Sciences, Northeastern University, Shenyang, Liaoning, China; ^2^Research Laboratory Center, Guizhou Provincial People's Hospital, Guizhou University, Guiyang, Guizhou, China; ^3^Department of Pathophysiology, Bengbu Medical College, Bengbu, Anhui, China; ^4^Department of Thoracic Surgery, The First Affiliated Hospital of Bengbu Medical College, Bengbu, Anhui, China

## Abstract

Glioblastoma is characterized as one of the deadliest cancers in humans. The survival time is not improved by standard treatment. Although immunotherapy has revolutionized cancer treatment, the current therapy targets for glioblastoma patients are not satisfied. We systematically analyzed the expression patterns, predictive values, and immunological characteristics of PTPN18 in glioblastoma. The independent datasets and functional experiments were employed to validate our findings. Our data showed that PTPN18 is potentially cancerogenic in glioblastoma with advanced grades and poor prognosis. High expression of PTPN18 correlated with CD8^+^ T cell exhaustion and immune suppression in glioblastoma. In addition, PTPN18 facilitates glioblastoma progression by accelerating glioma cell prefiltration, colony formation, and tumor growth in mice. PTPN18 also promotes cell cycle progression and inhibits apoptosis. Our results illustrate the characterization of PTPN18 in glioblastoma and highlight the potential value as an immunotherapeutic target for glioblastoma treatment.

## 1. Introduction

Glioblastoma multiforme (GBM) is the most common malignant tumor possessing up to 60% of primary brain tumors [1]. Despite improvements in independent and combinatorial treatments involving chemotherapy and radiotherapy, the survival rate of GBM remains very miserable [2]. The median survival is about 12 to 15 months after identification, and less than 3-7% of patients survive for more than five years [3]. Glioma cells conquer the immune system to reshape the microenvironments that benefit their development [4]. Since the unsatisfactory outcome after standard treatment, immunotherapy merits in-depth investigation as an additional option. Immune checkpoint inhibitors (such as nivolumab and pembrolizumab) have been used to treat GBM. Nevertheless, their efficiency has been discrepant and unpredictable in most GBM patients [5, 6]. Therefore, it is imperative to explore the effective therapeutic targets for GBM treatments.

PTPN18 is a member of the protein tyrosine phosphatase superfamily, which is involved in the progression and recurrence of multiple cancers [7]. The activity of PTPN18 is regulated by the different oxidation states of sulfur in its catalytic cysteine (C229 site), such as sulfenic acid (RSO^−^), sulfinic acid (RSO^2-^), or sulfonic acid (RSO^3-^) [8, 9]. Ectopic expression of PTPN18 facilitates cell growth and tumorigenesis of colorectal cancer. PTPN18 triggers MYC signaling by interacting with MYC and increases CDK4 protein expression in colorectal cancer [10]. A study also demonstrated that PTPN18 promotes endometrial cancer cell line proliferation and metastasis but inhibits their apoptosis [11]. We previously performed the pan-cancer analysis of classical protein tyrosine phosphatases [12] and confirmed that PTPN18 can inhibit breast cancer metastasis [13]. However, the potential functions and mechanisms of PTPN18 in glioblastoma remain unclear. In the present study, we explored the role of PTPN18 in glioblastoma progression with immune response. Our data revealed that PTPN18 exhibits potential cancerogenic properties in glioblastoma with a poor prognosis. Mechanistically, PTPN18 positively correlates with immune suppression and CD8^+^ T cell exhaustion and promotes glioblastoma progression by regulating the cell cycle and apoptosis. Together, PTPN18 may be a promising target for attenuating tumor immunosuppression for glioma treatment.

## 2. Materials and Methods

### 2.1. Data Source

The pan-cancer analyses were based on The Cancer Genome Atlas (TCGA) Research Network. RNA-seq data for the transcriptional expression of 33 types of cancer were downloaded using TCGAbiolinks R package [14]. The somatic mutation data were acquired from the MC3 project of TCGA PanCanAtla [15]. The copy number variations (CNVs) data were downloaded from Broad GDAC Firehose in January 2016 (https://gdac.broadinstitute.org/). GISTIC2 was employed to define the significant gain or loss in genomic regions [16]. The clinical data associated with TCGA patients were obtained from the published study [17] or downloaded using TCGAbiolinks R package. We downloaded the gene expression profiles from TCGA project and the Genotype-Tissue Expression (GTEx) project recomputed by the UCSC Xena project depending on a defined pipeline. The BH-adjusted *P* value < 0.05 was regarded as the differentially expressed genes in each cancer type. Validation data of glioma was required from the Chinese Glioma Genome Atlas (CGGA) and Gene Expression Omnibus (GEO) [18]. Other datasets were performed through the GlioVis dataset [19]. The cell line data were acquired from the Broad Institute Cancer Cell Line Encyclopedia (CCLE) and the Genomics of Drug Sensitivity in Cancer (GDSC) databases [20, 21].

### 2.2. Survival Analysis

The survival analysis was analyzed using the Kaplan–Meier method with the log-rank test by the survival R package. The cut-off point in each set was estimated using the survminer R package. *P* value < 0.05 was defined as significant.

### 2.3. Estimation of Immunological Characteristics in the TME

Immune cell infiltration was estimated using the published study based on CIBERSORT [22]. CIBERSORT is a deconvolution computation algorithm that can quantify hematopoietic cell composition based on the normalized gene expression matrix [23]. Since the cancer immunity cycle indicates what events are initiated to the killing of cancer cells [24], Xu et al. evaluated these events using a single sample gene set enrichment analysis (ssGSEA) based on bulk RNA-seq data [25]. T cell dysfunction and exclusion (TIDE) was used to predict cancer immunotherapy response [26]. Finally, we collected information on 129 immunomodulators involving antigen presentation, cell adhesion, coinhibitor, and costimulator from a previous study [27].

### 2.4. Functional Enrichment

Gene set enrichment analysis (GSEA) was used to analyze the potential functions of PTPN18 in glioma between the PTPN18 high and low groups [28]. An interactive network was constructed using Metascape to show the functional characteristics of PTPN18 and related genes [29].

### 2.5. Human Tissue Samples Collection

All human samples used in this study were collected from patients subjected to clinical surgery in the First Affiliated Hospital of Bengbu Medical College (Bengbu cohort) and stored at -80°C.

### 2.6. Immunohistochemistry

Target tissues were cut to 4 *μ*m thick, then deparaffinized, and rehydrated with xylene and graded alcohols (from 100% to 70%). After antigen retrieval with 5 mM citrate buffer, 3% H_2_O_2_ was used to inactivate endogenous peroxidase. The sections were blocked with goat serum for 30 min at room temperature and incubated with primary antibodies overnight at 4°C. The sections were washed with phosphate-buffered saline (PBS) three times and incubated with a biotinylated secondary antibody at room temperature for 2 h. Diaminobenzidine was used as a chromogen substrate. Finally, the sections were counterstained with hematoxylin. Antibody information is listed in Supplementary Table 1.

### 2.7. Cell Culture and Transfection

U118-MG and U-251-MG cell lines were cultured in Dulbecco's modified Eagle's medium (DMEM; HyClone, Thermo Fisher) supplemented with 10% fetal bovine serum (FBS; HyClone, Thermo Fisher) and 1% penicillin/streptomycin. U87-MG cell line was cultured in Eagle's minimum essential medium (EMEM; HyClone, Thermo Fisher) supplemented with 20% fetal bovine serum and 1% penicillin/streptomycin. All cell lines were maintained at 37°C in a humidified 5% CO_2_ chamber. Lipofectamine 3000 (Thermo Fisher) was applied to transient transfection for plasmids and siRNAs following the manufacturer's instruction.

### 2.8. Western Blotting

After 48 h transfection, cells were lysed on the ice, and equivalent amounts of denaturized proteins from each sample were separated using SDS-PAGE and then transferred to PVDF membranes. The membranes were incubated overnight with target primary antibodies at 4°C before blocking with 5% BSA in TBST at room temperature for 1 h. The membranes were washed with TBST three times, each for 5 min, followed by incubation with secondary antibodies at room temperature for 1 h. The blots were scanned, visualized, and analyzed using the ChemiDoc system (BioRad) and Image Lab software (BioRad). Antibody information is listed in Supplementary Table 1.

### 2.9. Colony Formation Assay

Cells were seeded in six-well plates at a density of 1000 cells. After two weeks of growth, colonies were fixed with paraformaldehyde for 30 min and marked with 0.1% crystal violet solution for 15 min. Finally, an optical microscope was used to counter the number of colonies.

### 2.10. Tumorigenesis in C57BL/6 Mice

Male mice (six weeks old, C57BL/6 mice) were acquired from Charles River (Beijing, China) and fed in the house in a pathogen-free condition. All procedures were approved by the Institutional Committee on Animal Care of Northeastern University. Murine glioma GL261 cells stably expressing PTPN18 and empty vector were injected subcutaneously into mice's right super lateral tissue (six mice per group, 6 × 10^7^ cells in serum-free DMEM). Mice were anatomized after 10 days. Western blot was used to detect the protein level of the target gene.

### 2.11. Cell Cycle and Apoptosis Analysis

Cell cycle and apoptosis were analyzed using propidium iodide (1 mg/ml) and ribonuclease-A (10 g/ml) (PI/RNase; BD Biosciences) and Annexin V/PI assay by flow cytometry (BD Biosciences, Franklin Lakes, NJ, USA) following manufacturer's instruction.

### 2.12. Statistical Analysis

Statistical analysis and graphical visualization were performed in R, version 4.0.0 (https://cran.r-project.org/). The student's *t*-test and the Wilcoxon rank-sum test were used to compare normally distributed and nonnormally distributed variables. The *P* values were two-sided and adjusted according to the Benjamini–Hochberg (BH) approach to control the false discovery rate (FDR). A BH-adjusted *P* value < 0.05 was considered statistically significant unless otherwise indicated.

## 3. Results

### 3.1. PTPN18 Exhibits Potential Cancerogenic Properties in Glioblastoma

**Figure 1:**
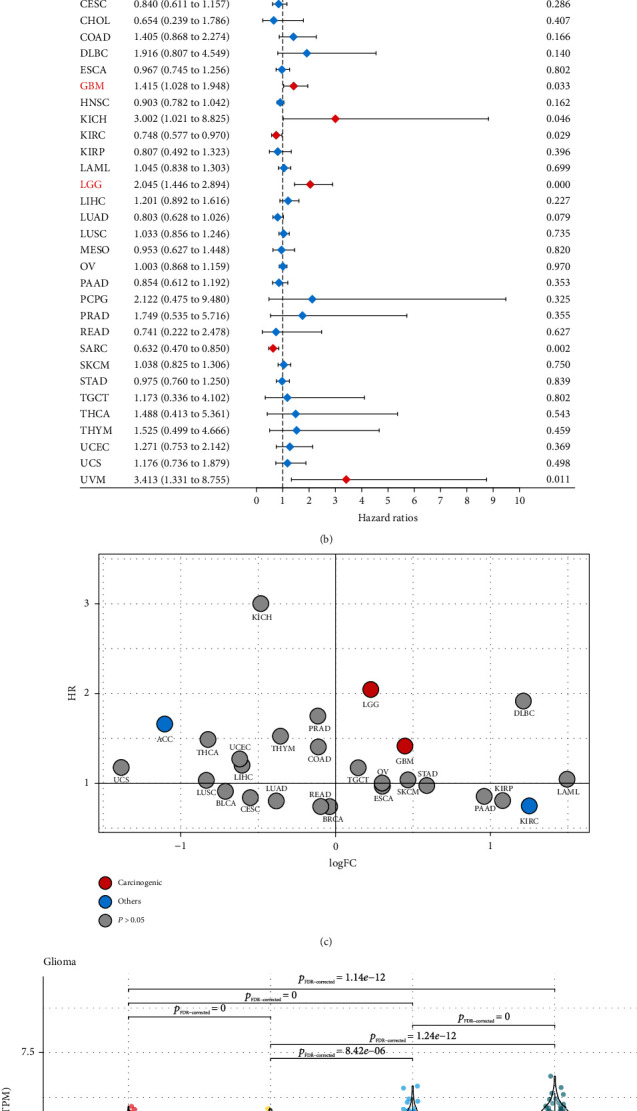
Potential cancerogenic properties of PTPN18 in human cancer. (a) The expression pattern of PTPN18 of pan-cancers in TCGA combined with GTEx. The thick line in the center of each box represents the median value. The bottom and top of the boxes are the 25th and 75th percentiles (interquartile range). The whiskers encompass 1.5 times the interquartile range. The statistical difference of two groups was compared through the Mann–Whitney *U* test. ^∗∗^*P* < 0.01; ^∗∗∗^*P* < 0.001; ^∗∗∗∗^*P* < 0.0001; ns: not significant. (b) Prognostic value of PTPN18 in different cancer types from TCGA datasets. (c) Potential cancerogenic properties of PTPN18 in cancers. (d) Expression of PTPN18 is associated with the grade in glioblastoma. (e) Kaplan–Meier curves for patients with high and low expression of PTPN18 in the glioblastoma.

We comprehensively analyzed the gene expression profiles from TCGA and GTEx, and observed that PTPN18 was highly expressed in some types of cancer, such as low-grade glioma (LGG) and GBM, compared with normal tissues (Figure 1(a)). The upregulation of PTPN18 was further validated in three independent glioma datasets (Figure S1A-C). PTPN18 was additionally expressed in various cancer cell lines, including glioma cell lines, according to the bulk data from the CCLE and GDSC datasets (Figure S1D-F). We also found that the expression of PTPN18 was significantly correlated with the stratification of glioma (Figure S1G-L). The pan-cancer expression pattern of PTPN18 provoked us to investigate its predictive value. The pan-cancer survival analyses were performed using the Cox regression model and log-rank test involving overall and cancer-specific survival. As shown in Figure 1(b), PTPN18 was an independent prognostic biomarker in some types of cancer. PTPN18 was associated with worse survival in LGG and GBM from TCGA and three independent glioma datasets (Figure 1(b); Figure S2A-C). Consistent with the overall survival, PTPN18 demonstrated a significant association with cancer-specific survival in seven types of cancer, including LGG and GBM (Figure S2D-E).

Based on the high expression and association with worse survival of PTPN18 in LGG and GBM (Figure 1(c)), we further explored the correlation of PTPN18 with clinicopathological characteristics. We found that patients with high expression levels of PTPN18 have an advanced grade and short survival time, presenting more aggressively than the low PTPN18 expression group (Figures 1(d) and 1(e)). These data suggested that PTPN18 might exclusively serve as a potential cancerogenic gene to promote glioma progression.

### 3.2. PTPN18 Shapes the Tumor Microenvironment in Glioblastoma

**Figure 2:**
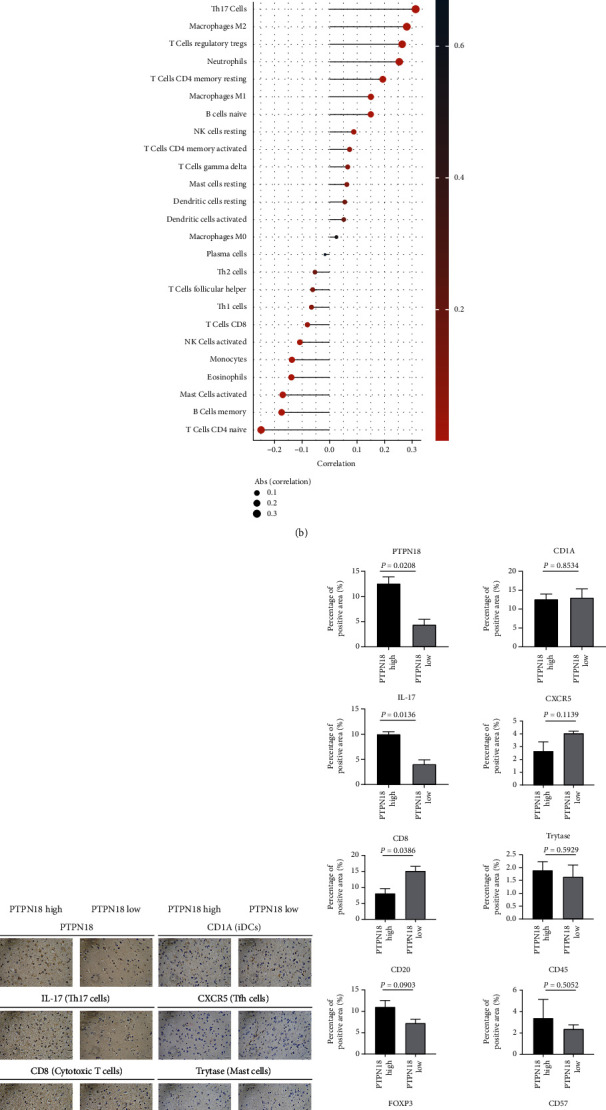
The effect of PTPN18 on immunological characteristics. (a) Deviations for the cancer immunity cycle between the PTPN18 high and low groups in glioblastoma. The thick line in the center of each box represents the median value. The bottom and top of the boxes are the 25th and 75th percentiles (interquartile range). The whiskers encompass 1.5 times the interquartile range. The statistical difference of two groups was compared through the Mann–Whitney *U* test. ^∗^*P* < 0.05, ^∗∗^*P* < 0.01; ^∗∗∗^*P* < 0.001; ^∗∗∗∗^*P* < 0.0001; ns: not significant. (b) Correlation between PTPN18 and infiltrated immune cells in glioma. (c) Representative IHC images of infiltrated immune cells in glioblastoma. (d) Quantification of (c) using Image-Pro Plus.

Cancer development and progression are associated with the immune cells present in the tumor microenvironment (TME) [30]. For the effective killing of cancer cells, a series of progressive events are initiated to activate an anticancer immune response referred to as the cancer immunity cycle [24]. In the high PTPN18 group, activities of some steps in the cycle were downregulated, including cancer antigen presentation (step 2), priming and activation (step 3), and killing of cancer cells (step 7) (Figure 2(a)). The depressed activities of these steps may affect the infiltration levels of specific types of immune cells to the TME. We, therefore, analyzed the correlation between the expression of PTPN18 and the infiltration of 25 immune cells in glioblastoma (Figure 2(b)) and validated the results using immunohistochemistry (Figures 2(c) and 2(d)). We found that PTPN18 were positively correlated with the infiltration of Th17 cells, M2 macrophages, and CD4^+^ memory T cells but negatively correlated with CD8^+^ T cells, B cells, and mast cells. To further explore the association between the expression of PTPN18 and antitumor immune response, we thoroughly inspected the immune-related genes with each cancer type. A general upregulation of the inhibitory immunomodulators was ascertained (Figure S3A).

### 3.3. PTPN18 Correlates with Immune Suppression and CD8^+^ T Cells Exhaustion in Glioblastoma

**Figure 3:**
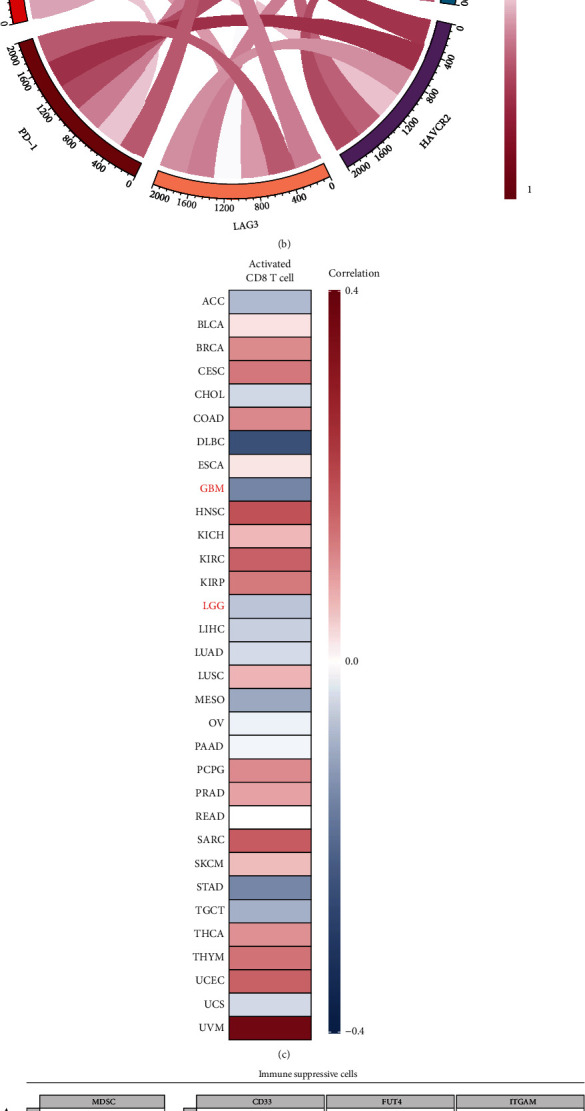
PTPN18 correlates with immune suppression and CD8+ T cell exhaustion in glioblastoma. (a) Correlation between PTPN18 and six immunosuppressive molecules across 33 types of cancer. The dots represent cancer types. The *Y*-axis represents the Spearman correlation, while the *X*-axis represents −log10*P*. Quadrant I: PTPN18 expression positively correlates with immunosuppressive genes, FDR < 0.05; quadrant II: PTPN18 expression positively correlates with immunosuppressive genes, FDR > 0.05; quadrant III: PTPN18 expression negatively correlates with immunosuppressive genes, FDR > 0.05; quadrant IV: PTPN18 expression negatively correlates with immunosuppressive genes, FDR < 0.05. (b) Correlation between PTPN18 and six immunosuppressive molecules in glioma. (c) Correlation of PTPN18 expression with activated CD8+ T cells in different types of cancer. (d) Correlation of PTPN18 expression with immunosuppressive cells (MDSC, TAM, Treg, and CAF) and their representative markers. TPM: transcripts per million.

The reduced proportion and defective function of CD8^+^ T cells are mainly attributed to the immunosuppressive genes and cells in the TME [31, 32]. We then explored the relationship between PTPN18 expression, immune checkpoints, and immunosuppressive cells involved in T cell exhaustion [33, 34]. We observed that PTPN18 expression was positively correlated with six immunosuppressive genes (PD-L1, PD-1, CTLA4, LAG3, HAVCR2, and CD244) in most cancers (Figure 3(a)), including glioma (Figure 3(b)). These immune checkpoints are involved in T cell activation and lead to the retrogression of T cell function [32]. In addition, we found that PTPN18 was significantly correlated with tumor mutation burden (TMB) and microsatellite instability (MSI) in several cancers, indicating that PTPN18 may imitate cancer immunogenicity in these cancers (Figure S3B-C).

Subsequently, we estimated the association of PTPN18 expression with the activation of CD8^+^ T cells and revealed that the infiltration of CD8^+^ T cells was adversely associated with PTPN18 in LGG (cor = −0.063, *P* < 0.05) and GBM (cor = −0.147, *P* < 0.05) (Figure 3(c)). Four immunosuppressive cells, myeloid-derived suppressor cell (MDSC), tumor-associated macrophage (TAM), cancer-associated fibroblasts (CAF), and regulatory T cell (Treg), can inhibit the infiltration of immune cells, especially CD8^+^ T cells, into the TME and suppress their functions within the tumor [34, 35]. PTPN18 expression was positively correlated with four immunosuppressive cells and their representative markers (Figure 3(d)).

### 3.4. Functional Analysis of PTPN18 in Glioblastoma

**Figure 4:**
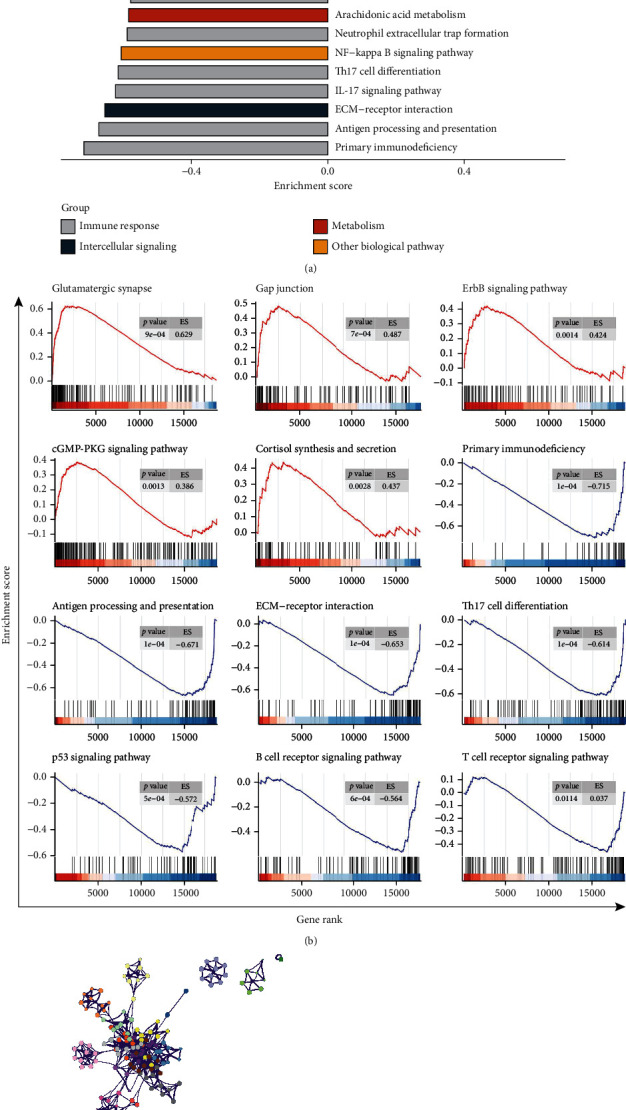
Functional enrichment of PTPN18 in glioblastoma. (a) Differences in pathway activities scored by GSEA between the PTPN18 high and low groups in TCGA dataset. (b) GSEA plot depicting representative pathways identified by GSEA between PTPN18 high and low groups in TCGA dataset. (c) Network of GO- and KEGG-enriched terms colored according to clusters. (d) Network of GO- and KEGG-enriched terms colored according to *P* values.

The enrichment analyses indicated that multiple cancer hallmark-related pathways varied notably between PTPN18 high and low groups, including immune response, intercellular signaling, metabolism, and other biological pathways (Figure 4(a)). Glutamatergic synapse, gap junction, ErbB signaling pathway, cGMP-PKG signaling pathway, and cortisol synthesis and secretion were significantly upregulated (Figure 4(b); FDR < 0.05). Primary immunodeficiency, antigen processing and presentation, ECM-receptor interaction, Th17 cell differentiation, p53 signaling pathway, B cell receptor signaling pathway, and T cell receptor signaling pathway were significantly downregulated (Figure 4(b); FDR < 0.05). Furthermore, the biological functions enrichment of PTPN18 and its related genes were explored using Metascape. Network of Gene Ontology (GO) and Kyoto Encyclopedia of Genes and Genomes (KEGG) terms colored according to cluster and *P* values were demonstrated (Figures 4(c) and 4(d)). Consistent with the GSEA results, PTPN18 was involved in anticancer immune response, which may advance the immunosuppressive microenvironment of glioma, as a clear example of inflammation-related cancer.

### 3.5. PTPN18 Promotes Glioblastoma Progression by Enhancing Immune Suppression

**Figure 5:**
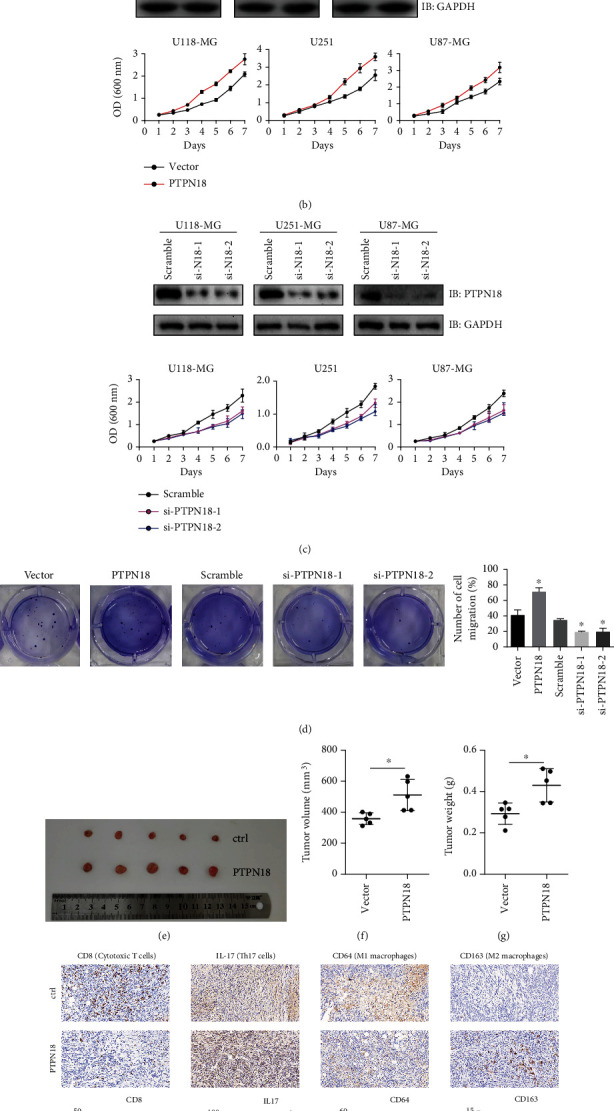
Functional validation of PTPN18 in glioma cells. (a) PTPN18 protein levels were detected in human glioma samples by western blot. (b) Growth curves demonstrate the effect of ectopic expression of PTPN18 on glioma cell lines. (c) Growth curves demonstrate the effect of PTPN18 knockdown on glioma cell lines. (d) Colony formation in the cells following the indicating treatment, ^∗^P < 0.05. (e) Tumors were harvested and photographed from C57 mice. (f, g) Final tumor volumes and weights were recorded and compared, ^∗^*P* < 0.05. (h) Representative IHC images of infiltrated immune cells in C57 mice, ^∗^*P* < 0.05, ^∗∗∗^*P* < 0.001.

To better address whether PTPN18 was correlated with glioma tumorigenesis, we analyzed the genomic alterations of PTPN18 and found that PTPN18 presented low mutational frequency across cancers with widespread CNV alterations (Figure S4). We further applied clinical specimens and observed that PTPN18 was significantly overexpressed in glioma samples contrasted with the paired adjacent samples at the protein level (Figure 5(a)). The ectopic expression and knockdown experiments were performed to evaluate the effect of PTPN18 on cell growth. We found that the overexpression of PTPN18 notably enhanced the growth velocity in different glioma cell lines (Figure 5(b)), while PTPN18 silencing inhibited cell growth (Figure 5(c)). Consistent with the above results, ectopic expression of PTPN18 promoted colony formation, while PTPN18 silencing inhibited colony formation (Figure 5(d)). To further elucidate the role of PTPN18 on glioblastoma progression, we evaluated the immune cell infiltration in C57BL/6 mice. We observed that glioblastoma cells stably expressing PTPN18 significantly promoted tumor growth (Figures 5(e)–5(g)). Moreover, PTPN18 prevented CD8^+^ T cells and M1 macrophages infiltrated into the lesion of tumor but accelerated Th17 cells and M2 macrophages infiltrating in to tumors (Figure 5(h)).

### 3.6. PTPN18 Promotes the Proliferation and Inhibits Apoptosis of Glioma Cell Lines

**Figure 6:**
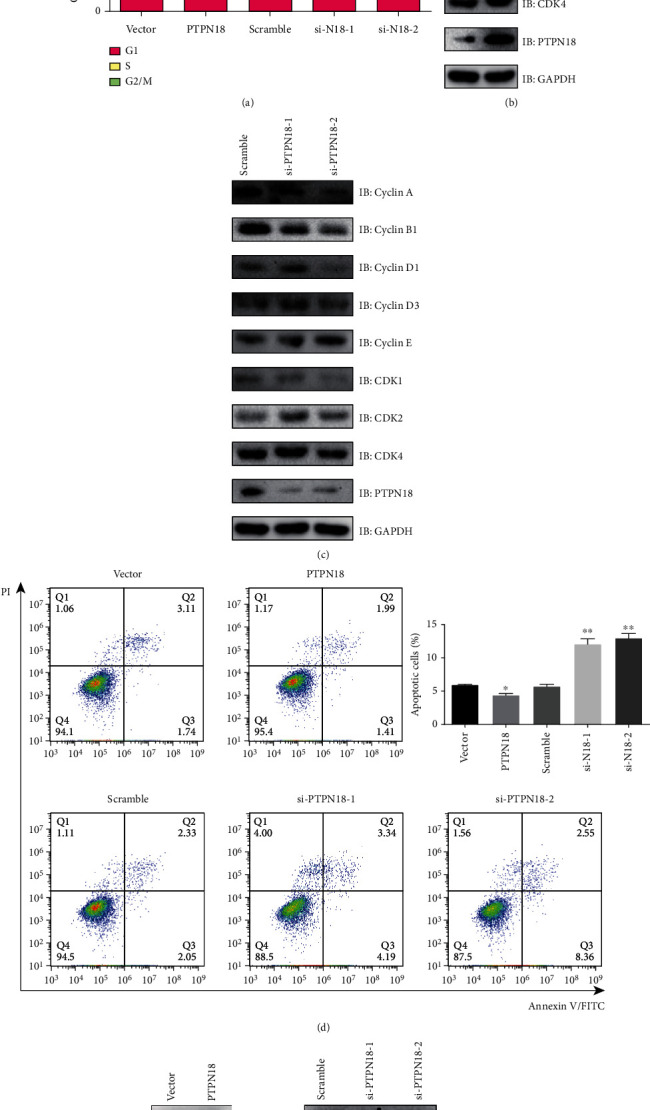
PTPN18 regulates cell cycle and apoptosis in glioma cells. (a) The effect of PTPN18 on cell cycle distribution. (b, c) Cell cycle-related genes were detected by western blot. (d) Flow cytometric analysis of early and late apoptotic cells with Annexin V and propidium iodide (PI). (e, f) Apoptosis-related genes were detected by western blot.

Cancer progression is closely connected with the regulation of cell cycle and apoptosis. We therefore performed cell cycle and apoptosis analysis on PTPN18 in glioma cells. Results showed that the proportion of cells in the G0/G1 phase decreased after PTPN18 overexpression but increased after PTPN18 knockdown (Figure 6(a)). Moreover, the proportion of cells in the S phase was significantly increased after PTPN18 expression but decreased after knockdown of PTPN18 (Figure 6(a)). To further explore the role of PTPN18 in cell cycle distribution, we examined the cell cycle-related proteins, such as Cyclin A, Cyclin B1, Cyclin D1, Cyclin D3, Cyclin E, CDK1, CDK2, and CDK4. Importantly, we observed that the expression of Cyclin A, Cyclin B1, and CDK1 was upregulated after PTPN18 overexpression but downregulated after PTPN18 knockdown (Figures 6(b) and 6(c)). However, the Cyclin D3, Cyclin E, and CDK2 expressions were downregulated after PTPN18 overexpression but upregulated after PTPN18 knockdown (Figures 6(b) and 6(c)). The expression of Cyclin D1 and CDK4 was not significantly different influenced by PTPN18 (Figures 6(b) and 6(c)). In sum, PTPN18 promotes cell cycle progression through modulating multiple cell cycle proteins.

To determine the influence of PTPN18 on cell apoptosis, an Annexin V-FITC/PI staining test was executed and evaluated by flow cytometry. We observed that PTPN18 overexpression slightly inhibited cell death more than the control. However, PTPN18 knockdown facilitated cell apoptosis (Figure 6(d)). The expression of antiapoptotic protein BCL2 was upregulated by PTPN18 overexpression but downregulated by PTPN18 knockdown. In addition, the expression of proapoptotic proteins BAX, BID, and BAK was downregulated by PTPN18 overexpression but upregulated by PTPN18 knockdown (Figures 6(e) and 6(f)). Together, these results suggest that PTPN18 is a potential oncogene in glioblastoma and may be a promising target for glioblastoma treatment.

## 4. Discussion

The crucial role of PTPN18 in tumorigenesis and abnormal PTPN18 expression in different cancer was described in previous studies [10, 11, 36]. In this study, we demonstrated the upregulation of PTPN18 in glioblastoma compared with normal tissue from TCGA and other independent datasets, which were validated using human specimens at the protein level. Moreover, PTPN18 significantly correlated with tumor progression and poor survival, indicating the potential cancerogenic properties of glioblastoma. Aberrantly expressed genes are accustomed to identifying molecular mechanisms of biological conditions [37]. Some studies reported that overexpression of PTPN18 promotes the cell growth and tumorigenesis of colorectal cancer [10] and accelerates endometrial cancer cell line proliferation and metastasis [11]. However, to our knowledge, the information regarding the effect of PTPN18 on glioblastoma progression is limited. We explored the association of the expression of PTPN18 with clinicopathological parameters and prognostic value to present more perspectives on the pathologic role of PTPN18 in glioblastoma progression. Our results showed that patients with higher PTPN18 expression have an advanced grade and correlate with the poor OS of glioblastoma. These data indicate that PTPN18 could serve as the prognostic biomarker for patients with glioblastoma and might be a promising target for glioblastoma treatment.

Brain tumors, including glioblastoma, are disreputable for triggering immunosuppression [38]. We explored the impacts of PTPN18 expression on the cancer immunity cycle. The cancer immunity cycle activities reflect the host's immune response to cancer and the products of complex immunomodulatory interactions in the TME. Our data showed that PTPN18 expression was involved in the cancer immunity cycle and negatively associated with multiple cycle stages. In particular, consisting of the inhibited activity of priming and activation in the PTPN18 high group, PTPN18 expression was adversely associated with PD1, PD-L1, and CTLA4, which are the inhibitors for priming and activation [24]. These immune checkpoints repress preexisting cancer immunity to avoid excessive immune response and lead to immune evasion. We further estimated the association of PTPN18 expression with immune infiltration and found that higher expression of PTPN18 correlated with reduced CD8^+^ T cells and poor prognosis in glioblastoma. The higher proportion of tumor-infiltrating CD8^+^ T cells improves survival and glioblastoma treatments [39, 40]. Therefore, we hypothesized that increased expression of PTPN18 led to glioblastoma progression by decreasing the infiltration of CD8^+^ T cells. Our study also indicated that PTPN18 expression was significantly associated with the immune checkpoints and immunosuppressive cells, such as PD-1, HAVCR2, TAM, MDSCs, and Tregs.

Furthermore, PTPN18 expression is significantly associated with IL-6 and IL-17 expression. Previous studies found that IL-6 induced the polarization of monocytes into TAM and the recruitment of MDSCs in the TME [41, 42]. TAM is the main contributor to systemic immunosuppression for GBM, and TAM-derived TGF*β* was the essential inducer for systemic immune tolerance [38]. IL-17 promotes PD-1 and HAVCR2 expression in CD8^+^ T cells in the TME [43]. TAM is thought to have both tumor-suppressing (M1 macrophages) and tumor-promoting (M2 macrophages) functions, giving them a nuanced role in carcinogenesis. Together, these data indicated that PTPN18 instigates glioblastoma progression by enhancing immunosuppression.

To further address whether PTPN18 was correlated with glioma tumorigenesis, we performed enrichment analyses of PTPN18 in glioblastoma. Our data showed that PTPN18 suppressed various immune response-related pathways and sustained the immunosuppressive microenvironment [44]. Functional experiments showed that ectopic PTPN18 promoted cell proliferation, colony formation, and tumor growth in nude mice, indicating the potential oncogenic role of PTPN18 in glioblastoma.

## 5. Conclusions

Overall, our results showed that the upregulation of PTPN18 in glioblastoma might effectively predict clinical prognosis. Moreover, this study demonstrated that the effect and mechanism of PTPN18 on promoting glioblastoma are mediated by reducing immune infiltration and enhancing immune suppression, indicating the potential value of targeting PTPN18 as an immunotherapy strategy for glioblastoma.

## Data Availability

All data used in this work can be acquired from the Chinese Glioma Genome Atlas (CGGA; http://www.cgga.org.cn/), the GlioVis dataset (http://gliovis.bioinfo.cnio.es/), the Gene Expression Omnibus (GEO; https://www.ncbi.nlm.nih.gov/geo/) under the accession number GSE4290, and the GDC portal (TCGA; https://portal.gdc.cancer.gov/).

## Supplementary Materials

Supplementary 1 Expression pattern of PTPN18 in glioblastoma.

Supplementary 2 Predictive analysis of PTPN18 for disease-specific survival in pan-cancers.

Supplementary 3 Correlations between PTPN18 and immunomodulators, TMB, and MSI in pan-cancers.

Supplementary 4 Genetic alterations of PTPN18 in pan-cancers.

Supplementary 5 Antibodies used in this study.
