# Supplementary material for: PTPN18 Serves as a Potential Oncogene for Glioblastoma by Enhancing Immune Suppression
Source: Oxid Med Cell Longev. 2023 Feb 15;2023:2994316. doi: 10.1155/2023/2994316 (PMC9950791; doi:10.1155/2023/2994316)
Supplement: Supplementary 1 — Expression pattern of PTPN18 in glioblastoma. [file 2994316.f1.pdf]

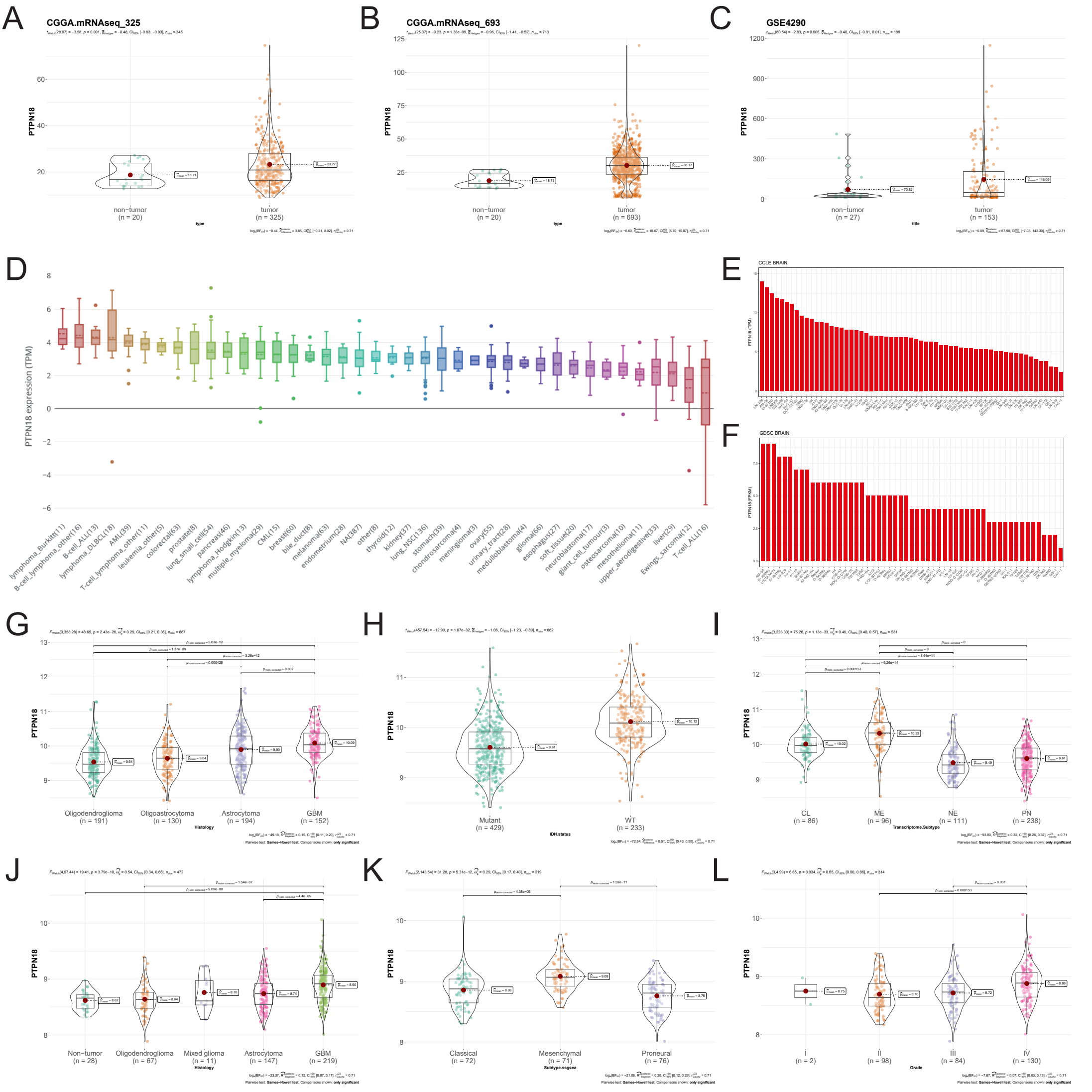

Fig. S1 Expression pattern of PTPN18 in glioblastoma. (A-C) PTPN18 expression between tumor and paired samples from CGGA and GEO dataset. (D) The cell line expression of PTPN18 in pan-cancer of CCLE. (E-F) The cell line expression of PTPN18 in glioblastoma of CCLE and GDSC. (G-L) PTPN18 expression in a different subgroup of glioblastoma from the GlioVis dataset.
