# Supplementary figures and images for: PTPN18 Serves as a Potential Oncogene for Glioblastoma by Enhancing Immune Suppression
Source: Oxid Med Cell Longev. 2023 Feb 15;2023:2994316. doi: 10.1155/2023/2994316 (PMC9950791; doi:10.1155/2023/2994316)

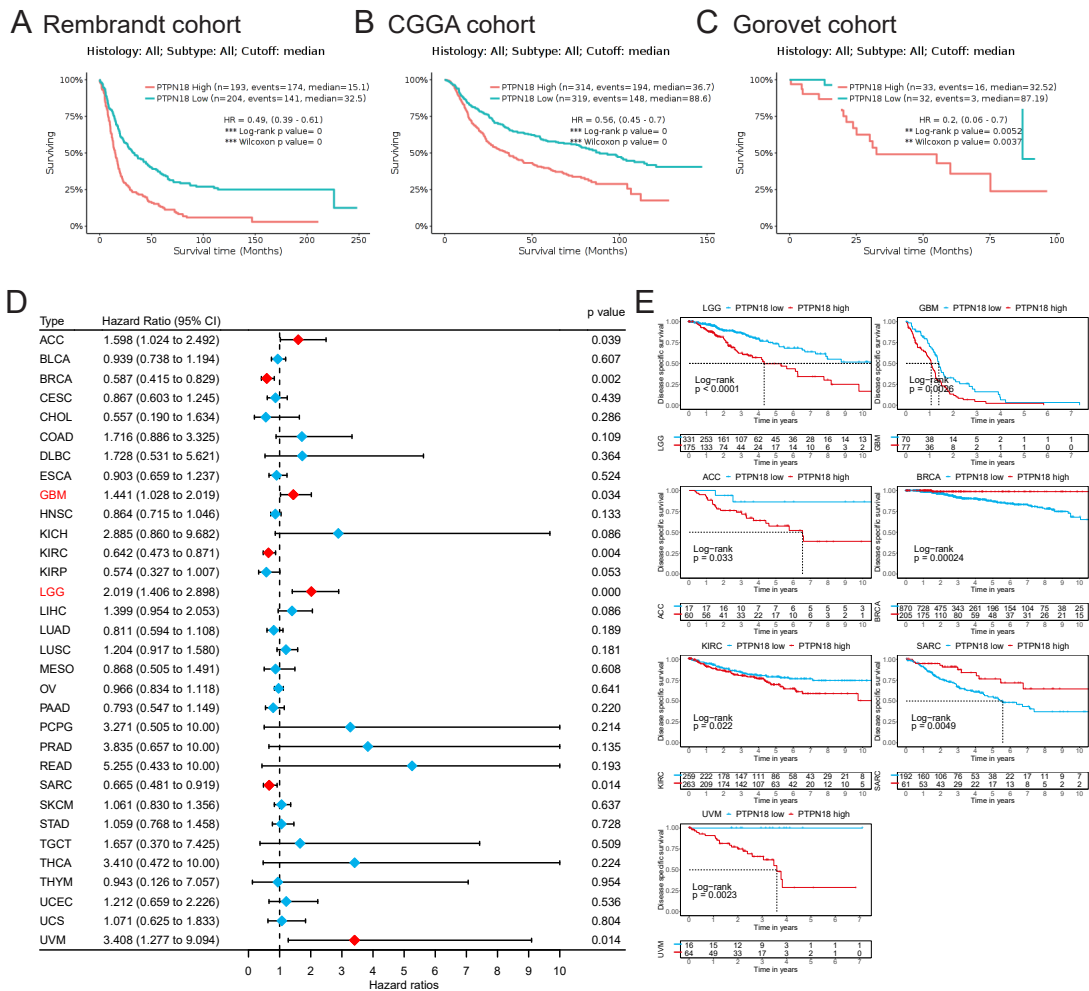

Supplement: Supplementary 2 — Predictive analysis of PTPN18 for disease-specific survival in pan-cancers. [file 2994316.f2.pdf]
