# Supplementary material for: PTPN18 Serves as a Potential Oncogene for Glioblastoma by Enhancing Immune Suppression
Source: Oxid Med Cell Longev. 2023 Feb 15;2023:2994316. doi: 10.1155/2023/2994316 (PMC9950791; doi:10.1155/2023/2994316)
Supplement: Supplementary 3 — Correlations between PTPN18 and immunomodulators, TMB, and MSI in pan-cancers. [file 2994316.f3.pdf]

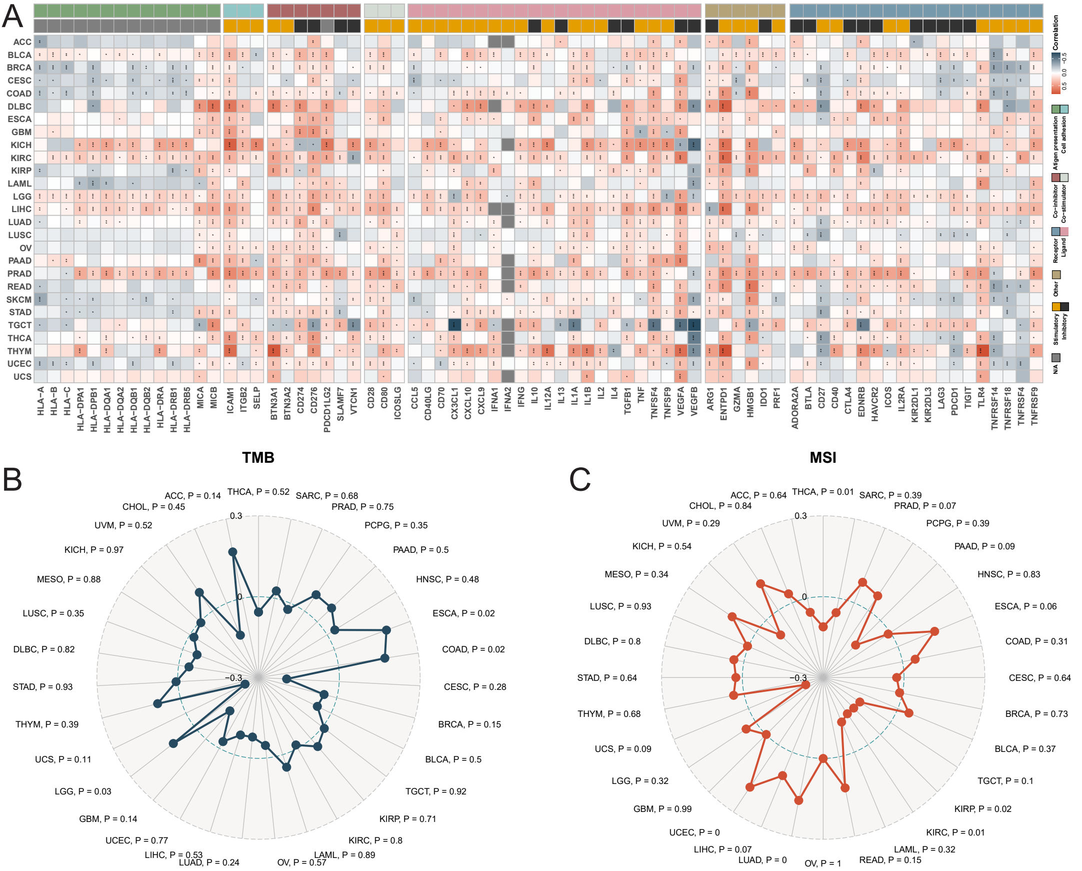

Fig. S3 Correlations between PTPN18, immunomodulators, TMB and MSI in pan-cancers. (A) Correlation between PTPN18 and immunomodulators. (B) Correlation between PTPN18 and TMB in pan-cancers. (C) Correlation between PTPN18 and MSI in pan-cancers.
