# Supplementary material for: PTPN18 Serves as a Potential Oncogene for Glioblastoma by Enhancing Immune Suppression
Source: Oxid Med Cell Longev. 2023 Feb 15;2023:2994316. doi: 10.1155/2023/2994316 (PMC9950791; doi:10.1155/2023/2994316)
Supplement: Supplementary 4 — Genetic alterations of PTPN18 in pan-cancers. [file 2994316.f4.pdf]

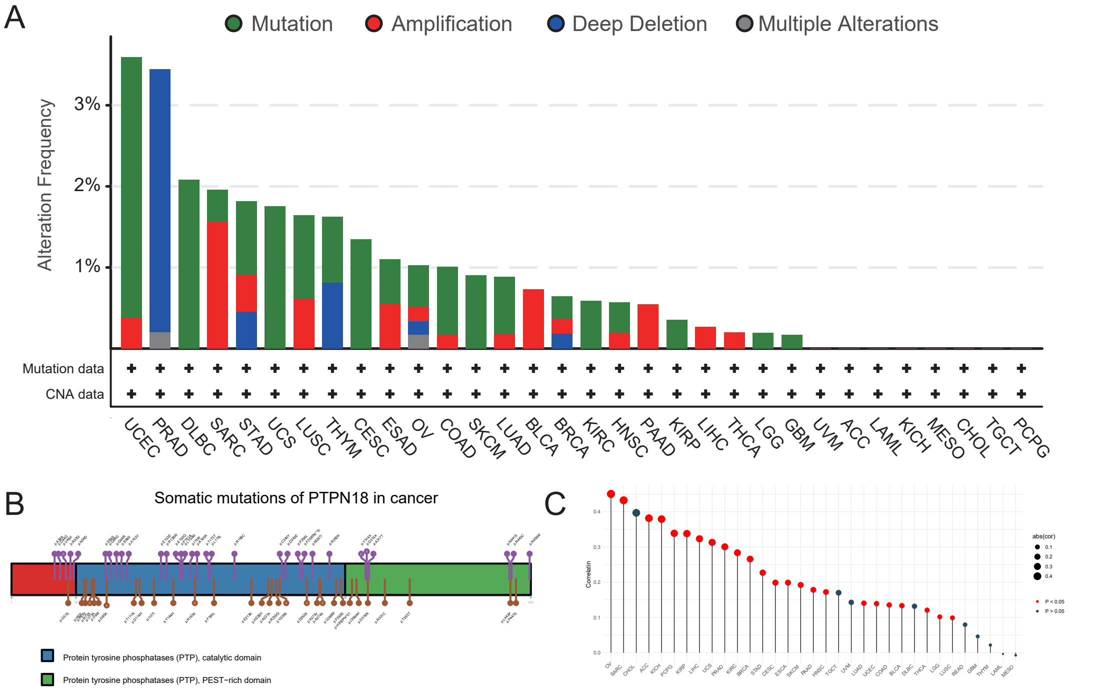

Fig. S4 Genetic alterations of PTPN18 in pan-cancers. (A) PTPN18 mutation frequency in multiple TCGA pan-cancer studies according to the cBioPortal database. (B) Gene-level summary of somatic mutations targeting PTPN18 in glioblastoma. (C) CNV alterations of PTPN18 across 33 types of cancers.
