# Supplementary material for: PTPN18 Serves as a Potential Oncogene for Glioblastoma by Enhancing Immune Suppression
Source: Oxid Med Cell Longev. 2023 Feb 15;2023:2994316. doi: 10.1155/2023/2994316 (PMC9950791; doi:10.1155/2023/2994316)
Supplement: Supplementary 5 — Antibodies used in this study. [file 2994316.f5.pdf]

Table S1. Antibodies used in this study.

Antibody	Source	Dilutions
α PTPN18	CST (8311)	WB (1:1000)
α CD1A	Proteintech (17325-1-AP)	IHC (1:200)
α IL-17	Abcam (ab79056)	IHC (1:200)
α CXCR5	Abcam (ab254415)	IHC (1:200)
α CD8	Proteintech (66868-1-Ig)	IHC (1:200)
α Trytase	Abcam (ab2378)	IHC (1:200)
α CD20	Proteintech (10252-1-AP)	IHC (1:200)
α CD45	Proteintech (60287-1-Ig)	IHC (1:200)
α FOXP3	Abcam (ab20034)	IHC (1:200)
α CD57	Proteintech (19401-1-AP)	IHC (1:200)
α CD64	Abcam (ab140779)	IHC (1:200)
α CD163	Abcam (ab79056)	IHC (1:200)
α Cyclin D1/Cyclin D3/CDK2/CDK	CST (9932)	WB (1:1000)
α Cyclin A2/Cyclin B1/Cyclin E2	CST (9870)	WB (1:1000)
α Bax/Bid/Bak	CST (9942)	WB (1:1000)
α Bcl2	CST (15071)	WB (1:1000)
α Flag	Absin (abs137958)	WB (1:1000)
α GAPDH	CST (2118)	WB (1:5000)
